# Clinical updates of approaches for biopsy of pulmonary lesions based on systematic review

**DOI:** 10.1186/s12890-018-0713-6

**Published:** 2018-09-03

**Authors:** Chuan-Jiang Deng, Fu-Qiang Dai, Kai Qian, Qun-You Tan, Ru-Wen Wang, Bo Deng, Jing-Hai Zhou

**Affiliations:** Department of Thoracic Surgery, Institute of Surgery Research, Daping Hospital, Army Medical University, Chongqing, 400042 People’s Republic of China

**Keywords:** Lung cancer, Percutaneous transthoracic needle biopsy, Electromagnetic navigation bronchoscopy, Endobroncheal ultrasonography, Circulating tumor cell

## Abstract

**Background:**

Convenient approaches for accurate biopsy are extremely important to the diagnosis of lung cancer. We aimed to systematically review the clinical updates and development trends of approaches for biopsy, i.e., CT-guided PTNB (Percutaneous Transthoracic Needle Biopsy), ENB (Electromagnetic Navigation Bronchoscopy), EBUS-TBNA (Endobroncheal Ultrasonography-Transbronchial Needle Aspiration), mediastinoscopy and CTC (Circulating Tumor Cell).

**Methods:**

Medline and manual searches were performed. We identified the relevant studies, assessed study eligibility, evaluated methodological quality, and summarized diagnostic yields and complications regarding CT-guided PTNB (22 citations), ENB(31 citations), EBUS-TBNA(66 citations), Mediastinoscopy(15 citations) and CTC (19 citations), respectively.

**Results:**

The overall sensitivity and specificity of CT-guided PTNB were reported to be 92.52% ± 3.14% and 97.98% ± 3.28%, respectively. The top two complications of CT-guided PTNB was pneumothorax (946/4170:22.69%) and hemorrhage (138/1949:7.08%). The detection rate of lung cancer by ENB increased gradually to 79.79% ± 15.34% with pneumothorax as the top one complication (86/1648:5.2%). Detection rate of EBUS-TBNA was 86.06% ± 9.70% with the top three complications, i.e., hemorrhage (53/8662:0.61%), pneumothorax (46/12432:0.37%) and infection (34/11250:0.30%). The detection rate of mediastinoscopy gradually increased to 92.77% ± 3.99% with .hoarseness as the refractory complication (4/2137:0.19%). Sensitivity and specificity of CTCs detection by using PCR (Polymerase Chain Reaction) were reported to be 78.81% ± 14.72% and 90.88% ± 0.53%, respectively.

**Conclusion:**

The biopsy approaches should be chosen considering a variety of location and situation of lesions. CT-guided PTNB is effective to reach lung parenchyma, however, diagnostic accuracy and incidence of complications may be impacted by lesion size or needle path length. ENB has an advantage for biopsy of smaller and deeper lesions in lung parenchyma. ENB plus EBUS imaging can further improve the detection rate of lesion in lung parenchyma. EBUS-TBNA is relatively safer and mediastinoscopy provides more tissue acquisition and better diagnostic yield of 4R and 7th lymph node. CTC detection can be considered for adjuvant diagnosis.

## Background

Lung cancer is the most frequently diagnosed cancer and continues to be the leading cause of cancer mortality among both males and females [1]. The 5-year survival rate of lung cancer is only 18%, largely due to late-stage diagnosis [1]. Thus, early diagnosis is especially critical to improve long-term survival. Biopsy is important for identification and confirmation of lung cancer. In clinical practice, conventional flexible bronchoscopy is supposed to be difficult for biopsy of small lesions in lung parenchyma or mediastinum. Therefore, we focused on the following approaches for biopsy according to a variety of lesion location in lung parenchyma, i.e., CT-guided PTNB(Percutaneous Transthoracic Needle Biopsy), ENB (Electromagnetic Navigation Bronchoscopy), EBUS-TBNA (Endobroncheal Ultrasonography-Transbronchial Needle Aspitation) and mediastinoscopy. Furthermore, the studies regarding liquid biopsies, e.g., CTC (Circulating Tumor Cell) detection are timely and hot, and warrant to be systematically reviewed.

Therefore, we evaluated the published studies in the last 20 years which focused on CT-guided PTNB, ENB, EBUS-TBNA, mediastinoscopy and CTC, aiming to reveal the clinical updates, development trends, detection rates and complications.

## Methods

We used systematic review to identify relevant studies, assess study eligibility, evaluate methodological quality, and summarize findings regarding postoperative clinical outcomes. Medline and manual searches were performed by investigators CJD and FQD independently and jointly to identify all published articles in English journals from January 1, 2000 to November 9, 2017 that addressed the issues regarding detection of lung cancers by using CT-guided PTNB, ENB, EBUS-TBNA, mediastinoscopy and CTCs, respectively. The Medline search was done on PubMed (http://www.ncbi.nlm.nih.gov). The search strategies and yielded citations were shown in Tables 1 and 2, respectively. Investigators CJD and FQD performed the actual search and data abstraction.

**Table 1:** Data sources and searches regarding Clinical updates of approaches for biopsy

Methods	Search term	Period	Additional filters	Citation number after filtration	Citation number after Manual verification
CT-guided PTNB	ct guided transthoracic needle biopsy[All Fields] AND lung neoplasms[MeSH Terms]	From January 1, 2000 To November 9, 2017	English, humans without review	106	22
ENB	‘electromagnetic navigation bronchoscopy (ENB)’[All Fields]	From January 1, 2000 To November 9, 2017	English, humans without review	91	31
EBUS-TBNA	EBUS[All Fields] AND “lung neoplasms” [MeSH Terms]	From January 1, 2000 To November 9, 2017	English, humans without review	613	66
Mediastinoscopy	Mediastinoscopy[Mesh Terms] AND “lung neoplasms”[MeSH Terms]	From January 1, 2000 To November 9, 2017	English, humans without review	333	15
CTC	‘Neoplastic Cells, Circulating’[Mesh Terms] AND “lung neoplasms”[MeSH Terms]	From January 1, 2000 To November 9, 2017	English, humans without review	459	19

**Table 2:** Information of yielded citations regarding approaches for biopsy

PMID	Year	Method	Corresponding author	Cases	Diagnostic sensitivity
28,415,930	2017	CT-guided PTNB	Feride Fatma Go¨rgu¨lu¨	65	90.80%
28,063,634	2016	CT-guided PTNB	C. Fontaine-Delaruelle	929	N/A
26,980,483	2016	CT-guided PTNB	Mickey Sachdeva	203	N/A
26,397,325	2015	CT-guided PTNB	M. Petranovic	52	N/A
26,110,775	2015	CT-guided PTNB	Wen Yang	311	77%
25,903,714	2015	CT-guided PTNB	Matthew Koslow	181	94.40%
25,816,042	2015	CT-guided PTNB	Fabio Pagni	N/A	97.60%
25,662,328	2015	CT-guided PTNB	Anna Galluzzo	23	87%
25,569,025	2015	CT-guided PTNB	Sébastien Couraud	980	90%
25,051,977	2014	CT-guided PTNB	Tingyang Hu	341	N/A
24,581,458	2014	CT-guided PTNB	Jeffrey S. Klein	32	N/A
24,475,839	2014	CT-guided PTNB	Chang Min Park	1108	97%
25,763,320	2014	CT-guided PTNB	Sanjay Piplani	74	95.94%
23,510,132	2013	CT-guided PTNB	Antonio Bugalho	123	N/A
23,079,048	2013	CT-guided PTNB	Yi-Ping Zhuang	102	96.10%
22,951,610	2012	CT-guided PTNB	Ragulin IuA	107	N/A
22,124,475	2012	CT-guided PTNB	Yeun-Chung Chang	55	N/A
21,537,657	2012	CT-guided PTNB	Lu CH	89	91.50%
21,098,171	2010	CT-guided PTNB	Hye Sun Hwang	27	94%
15,246,522	2004	CT-guided PTNB	Ohno Y	N/A	96.90%
14,595,149	2003	CT-guided PTNB	Stephen T. Kee	846	96%
14,595,149	2003	CT-guided PTNB	Stephen T. Kee	846	92%
12,118,196	2002	CT-guided PTNB	Adnan Yilmaz	294	88%
28,410,635	2017	ENB	Christopher W. Towe	341	N/A
27,623,421	2017	ENB	Michael Chacey	31	96.80%
28,459,951	2017	ENB	Kongjia Luo	24	100.00%
28,449,489	2017	ENB	Hiran C. Fernando	17	79.00%
28,399,830	2017	ENB	Erik E. Folch	1000	N/A
26,944,363	2016	ENB	Mohammed Al-Jaghbeer	92	60.00%
27,157,054	2016	ENB	Arjun Pennathur	29	100.00%
27,424,820	2016	ENB	Fumihiro Asano	932	71.00%
25,849,298	2015	ENB	Demet Karnak	44	72.80%
25,590,477	2015	ENB	Mark R. Bowling	107	73.60%
24,739,685	2014	ENB	Nima Nabavizadeh	31	N/A
24,401,166	2014	ENB	Gregoire Gex	971	64.90%
23,440,066	2013	ENB	Demet Karnak	76	89.50%
24,323,803	2013	ENB	Rana S Hoda	40	94.00%
23,649,436	2013	ENB	*M. Patricia* Rivera	932	71.00%
22,391,437	2012	ENB	B.Lamprecht	112	83.90%
22,277,964	2012	ENB	Daryl Phillip Pearlstein	104	85.00%
23,207,529	2012	ENB	Christopher R Dale	100	N/A
23,207,349	2012	ENB	Kyle R. Brownback	55	74.50%
23,207,460	2012	ENB	Kurt W. Jensen	92	65.00%
23,169,081	2011	ENB	Amit K. Mahajan	49	77.00%
20,850,809	2010	ENB	Carsten Schroeder	52	N/A
20,802,352	2010	ENB	Felix J. F. Herth	25	80.00%
20,435,658	2010	ENB	Luis M. Seijo	51	67.00%
19,648,733	2010	ENB	med. Ralf Eberhardt	54	75.50%
19,546,519	2009	ENB	Jean-Michel Vergnon	54	71.40%
17,400,670	2007	ENB	Armin Ernst	92	67.00%
17,360,724	2007	ENB	C-H. Marquette	40	62.50%
17,532,538	2007	ENB	Motoko Tachihara	94	62.50%
17,379,850	2007	ENB	Armin Ernst	120	59.00%
16,873,767	2006	ENB	Thomas R. Gildea	60	74.00%
29,054,229	2017	EBUS-TBNA	Chen-Yoshikawa	413	N/A
27,710,975	2016	EBUS-TBNA	Fumihiro Tanaka	20	75.00%
27,435,209	2016	EBUS-TBNA	João Pedro Steinhauser Motta	84	61.00%
27,409,724	2015	EBUS-TBNA	Whittney A. Warren	333	98.86%
27,150,855	2016	EBUS-TBNA	Sang-Won Um	161	94.00%
26,656,954	2015	EBUS-TBNA	Baijiang Zhang	114	81.20%
26,545,094	2015	EBUS-TBNA	Wen-Chien Cheng	2527	N/A
26,386,084	2015	EBUS-TBNA	Massimo Barberis	291	95.53%
26,176,519	2015	EBUS-TBNA	Sebastián Fernández-Bussy	145	91.17%
25,611,227	2015	EBUS-TBNA	Sang-Won Um	138	92.90%
25,584,815	2014	EBUS-TBNA	Roberto F. Casal	220	N/A
25,170,748	2014	EBUS-TBNA	Andrew R.L. Medford	70	90.00%
25,149,044	2014	EBUS-TBNA	Masato Shingyoji	113	88.40%
24,930,616	2014	EBUS-TBNA	Masahide Oki	150	89%
24,853,017	2014	EBUS-TBNA	Yasushi Murakami	100	97.00%
24,419,182	2013	EBUS-TBNA	Paul F. Clementsen	76	88.16%
24,340,058	2013	EBUS-TBNA	Takayuki Shiroyama	178	73.60%
24,238,520	2014	EBUS-TBNA	Zhao H	66	89.40%
24,172,712	2013	EBUS-TBNA	Kang HJ	74	93.20%
24,125,976	2013	EBUS-TBNA	Ozgül MA	40	94.70%
24,079,724	2013	EBUS-TBNA	Lonny Yarmus	85	100.00%
24,075,565	2013	EBUS-TBNA	Yinin Hu	231	90.00%
23,994,976	2013	EBUS-TBNA	Sang-Won Um	42	95.30%
23,953,728	2013	EBUS-TBNA	Konstantinos Syrigos	981	76.20%
23,723,003	2013	EBUS-TBNA	Guo-liang Xu	128	93.00%
23,663,438	2013	EBUS-TBNA	Fumihiro Asano	7345	N/A
23,639,784	2013	EBUS-TBNA	Riccardo Inchingolo	662	77.00%
23,609,248	2013	EBUS-TBNA	Christian B. Gindesgaard	116	87.00%
23,609,243	2013	EBUS-TBNA	Hammad A. Bhatti	13	94.00%
23,571,718	2013	EBUS-TBNA	Masahide Oki	108	88.00%
23,549,813	2013	EBUS-TBNA	Sang-Won Um	37	86.40%
23,245,441	2012	EBUS-TBNA	Kazuhiro Yasufuku	438	96.50%
23,117,878	2014	EBUS-TBNA	George A. Eapen	1317	N/A
24,632,834	2014	EBUS-TBNA	Sang-Won Um	44	79.00%
24,603,902	2013	EBUS-TBNA	Moishe Liberman	161	72.00%
22,219,613	2012	EBUS-TBNA	Sang-Won Um	151	91.60%
22,154,791	2011	EBUS-TBNA	Benjamin E. Lee	73	95.00%
21,963,329	2011	EBUS-TBNA	Kazuhiro Yasufuku	153	81.00%
21,792,077	2011	EBUS-TBNA	Sam M. Janes	161	87.00%
21,718,857	2011	EBUS-TBNA	Alexander Chen	50	81.00%
21,651,742	2011	EBUS-TBNA	Shahab Nozohoo	243	66.00%
21,592,457	2010	EBUS-TBNA	Kazuhiro Yasufuku	450	93.10%
20,819,667	2010	EBUS-TBNA	Tian Q	33	69.70%
20,740,503	2010	EBUS-TBNA	Qing Kay Li	47	89.50%
20,609,781	2010	EBUS-TBNA	Kazuhiro Yasufuku	N/A	96.40%
20,372,904	2010	EBUS-TBNA	J. Eckardt	308	72.00%
20,138,390	2010	EBUS-TBNA	Bin Hwangbo	126	97.20%
20,037,856	2010	EBUS-TBNA	Sökücü SN	N/A	88.20%
20,022,759	2010	EBUS-TBNA	Artur Szlubowski	61	67.00%
19,890,836	2009	EBUS-TBNA	Wei Sun	64	88.90%
19,789,210	2009	EBUS-TBNA	Andrew RL Medford	54	89.00%
19,699,917	2009	EBUS-TBNA	Sebastien Gilbert	172	86.60%
19,590,457	2009	EBUS-TBNA	Armin Ernst	N/A	91.00%
19,502,074	2009	EBUS-TBNA	Henrik Ømark Petersen	157	85.00%
19,447,014	2009	EBUS-TBNA	Devanand Anantham	N/A	90.00%
19,371,395	2008	EBUS-TBNA	David Fielding	68	94.00%
19,068,672	2008	EBUS-TBNA	Marie-Paule Jacob-Ampuero	48	77.00%
18,952,453	2009	EBUS-TBNA	Jarosław Kuzdza	226	89.00%
18,263,680	2007	EBUS-TBNA	Armin Ernst	100	89.00%
17,916,175	2008	EBUS-TBNA	Mariko Siyue Koh	38	62.00%
17,379,850	2006	EBUS-TBNA	Armin Erns	120	69.00%
17,035,455	2007	EBUS-TBNA	Meng-Chih Lin	151	73.80%
16,963,667	2006	EBUS-TBNA	Takehiko Fujisawa	102	92.30%
16,807,262	2005	EBUS-TBNA	F.J.F. Herth	100	92.30%
16,171,897	2005	EBUS-TBNA	Takehiko Fujisawa	105	94.60%
27,385,137	2016	Mediastinoscopy	Necati C¸itak	261	96.00%
27,385,137	2016	Mediastinoscopy	Necati C¸itak	187	95.00%
24,751,152	2014	Mediastinoscopy	Benjamin Wei	721	87.10%
23,778,084	2013	Mediastinoscopy	Akif Turna	344	92.20%
23,778,084	2013	Mediastinoscopy	Akif Turna	89	96.60%
23,008,924	2012	Mediastinoscopy	Ashutosh Chauhan	39	87.50%
22,219,461	2012	Mediastinoscopy	Carme Obiolsa	221	95.00%
21,601,176	2011	Mediastinoscopy	Young Mog Shim	521	95.90%
20,417,780	2010	Mediastinoscopy	Yaron Shargall	104	98.90%
20,417,780	2010	Mediastinoscopy	Yaron Shargall	396	97.20%
18,520,794	2008	Mediastinoscopy	Armin Ernst	66	78.00%
18,687,697	2008	Mediastinoscopy	Elias A. Karfis	139	88.40%
18,054,494	2007	Mediastinoscopy	Gunda Leschber	377	87.90%
12,842,542	2003	Mediastinoscopy	Jèrôme Mouroux	154	98.00%
12,683,545	2003	Mediastinoscopy	Didier Lardinois	195	95.60%
11,321,666	2001	Mediastinoscopy	Reidar Grénman	249	84.30%
26,913,536	2016	CTC	María Jose Serrano	56	51.80%
26,951,195	2016	CTC	Noriyoshi Sawabata	23	30.40%
27,206,795	2016	CTC	Binlei Liu	40	55.00%
27,206,795	2016	CTC	Binlei Liu	40	75.00%
25,996,878	2015	CTC	Wei Li	169	23.70%
25,678,504	2014	CTC	Mario Santini	16	89.00%
23,861,795	2013	CTC	Viswam S. Nair	43	60.47%
21,098,695	2011	CTC	Paul Hofman	208	49.00%
21,215,651	2011	CTC	Noriyoshi Sawabata	75	69.33%
21,683,606	2011	CTC	Renato Franco	45	23.90%
21,128,227	2010	CTC	Paul Hofman	210	39.00%
21,128,227	2010	CTC	Paul Hofman	210	50.00%
20,471,712	2010	CTC	Chul-Woo Kim	61	42.60%
20,471,712	2010	CTC	Chul-Woo Kim	61	36.10%
19,887,487	2009	CTC	Fumihiro Tanaka	125	71.00%
18,514,066	2008	CTC	Yan-hui Yin	134	84.30%
18,606,477	2008	CTC	Shang-mian Yie	67	38.80%
17,554,991	2007	CTC	Noriyoshi Sawabata	9	11.10%
16,642,481	2006	CTC	Inn-Wen Chong	100	90.00%
15,801,980	2005	CTC	Katharina Pachmann	29	86.21%
12,167,790	2002	CTC	Michio Ogawa	57	38.60%

### Data abstraction

From the eligible articles, investigators CJD and FQD reviewed the following information, i.e., PMID, year of publication, study design, number of patients, average age of patients, nodules size and location, operation time, biomarkers for detection, diagnostic sensitivity, relative complication morbidity, treatment of complications, outcome and follow-up period.

### Statistical analysis

The association between detection rate of ENB and nodule size, number of cases, operation time, average age of patients, sex, and mean distance of the lesions from the pleura was performed using Pearson’s correlation analysis. The impact of nodule location on detection rate of ENB was analyzed by using ANOVA analysis. The association between morbidity of pneumothorax following ENB and nodule size was performed using Pearson’s correlation analysis. The analyses were performed using SPSS Version 11.0 software for Windows (SPSS, Inc., Chicago, IL, USA). *P* < 0.05 (two-sided) was considered to indicate a statistically significant difference.

## Results

### CT-guided PTNB: Biopsy of lesion in lung parenchyma mapped on CT images

In last 20 years, the overall sensitivity, specificity, and accuracy of CT-guided PTNB were 92.52 ± 3.14%, 97.98 ± 3.28%, and 92.28% ± 5.40%, respectively. The top two complications of CT-guided PTNB were pneumothorax (1111/4822:23.04%) and hemorrhage (287/3503:8.19%), respectively. Two cases with severe complications were reported [2, 3]. Bronchial artery embolization was performed in one patient due to massive hemoptysis [3]. The other one suffered from cardiopulmonary arrest leading to death [2].

Diagnostic accuracy and incidence of complications seemed to be decreased [3–5] and increased [2–9], respectively, by smaller lesion size or longer needle path length (*P <* 0.05).

### ENB: Biopsy of lesion in lung parenchyma and mediastinal area

**Fig. 1:**
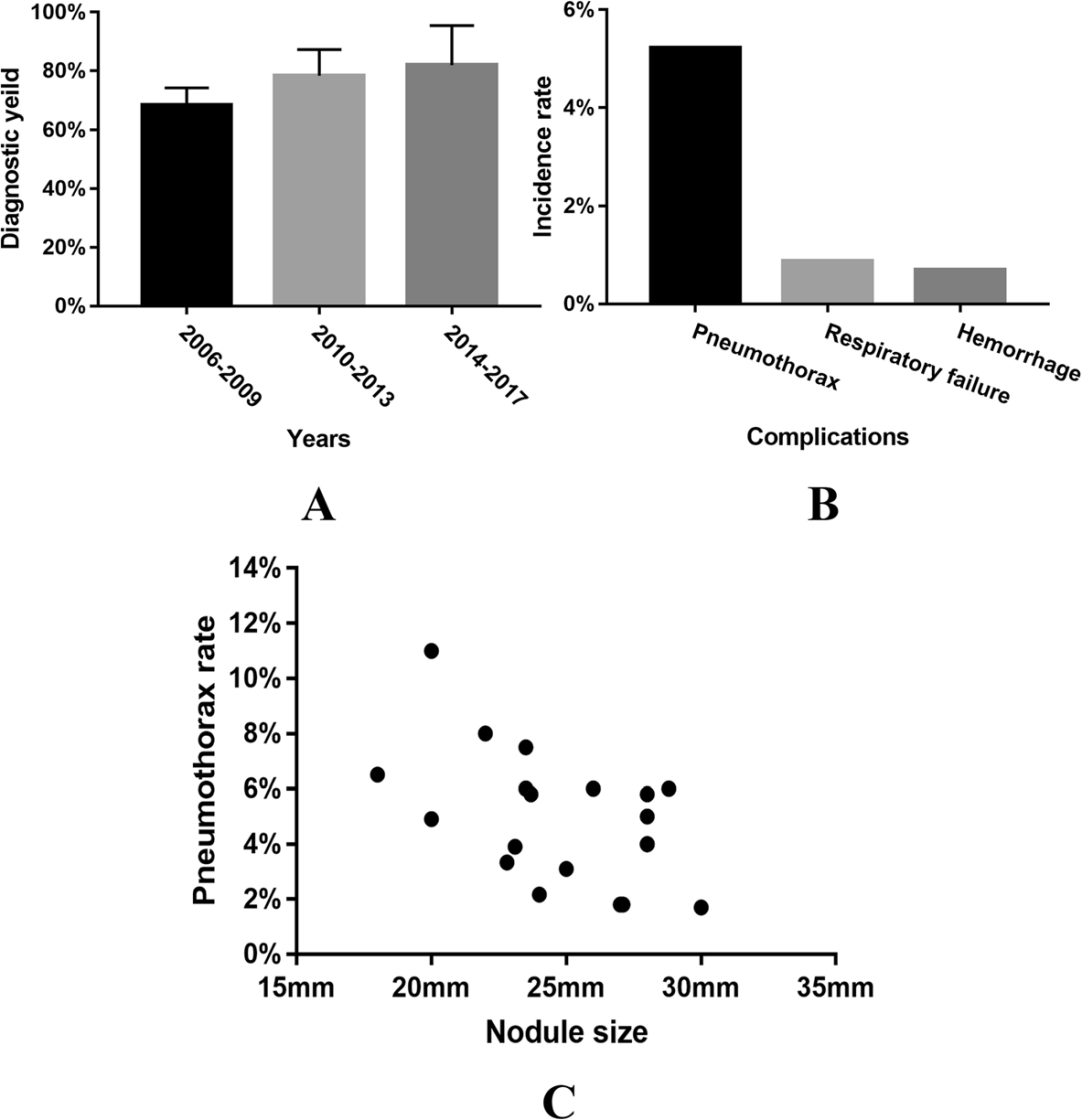
Analysis of clinical points regarding ENB. **a** Correlation between detection rate and publication time showing the detection rate increased gradually. **b** Pneumothorax was the top one complication following ENB (86/1648:5.2%). **c** The morbidity of pneumothorax was significantly negatively correlated with nodule size (*R* = −0.512, *P* = 0.018)

The detection rate of lung cancer by ENB increased gradually (Fig. 1a) and was recently reported to be 96.8% [10]. There seemed to be no significant correlation between detection rate and number of cases, average age of patients, sex, nodule size, lobar location of nodule, mean distance from pleura to nodule and operation time. As shown in Fig. 1b, pneumothorax was the top one complication following ENB (86/1648:5.2%). In 86 pneumothorax cases, 34 cases (34/86) were administrated with closed drainage [10–21], and one case (1/86) was managed with manual aspiration and observation [19]. The other 51 cases with mild pneumothorax were discharged for rehabilitation. Intriguingly, the incidence of pneumothorax was significantly negatively correlated with nodule size (*R* = − 0.512, *P* = 0.018, Fig. 1c). The three hemorrhage cases were observed carefully without further intervention and were discharged for rehabilitation [16, 22]. Three cases of respiratory failure were reported without detailed depiction [16]. There were no ENB related death [10–30]. ENB plus EBUS imaging seemed to yield a higher detection rate as compared with sole use of ENB (59% vs. 88% [20] and 71.42% vs. 73.07% [11]). Surprisingly, studies combining fluoroscopy with ENB to confirm navigation success reported lower diagnostic yields (56.3 vs. 69.2% without fluoroscopy, *p* = 0.006) [31].

### EBUS-TBNA: Biopsy of lesion in subcarinal and bilateral hilar area

**Fig. 2:**
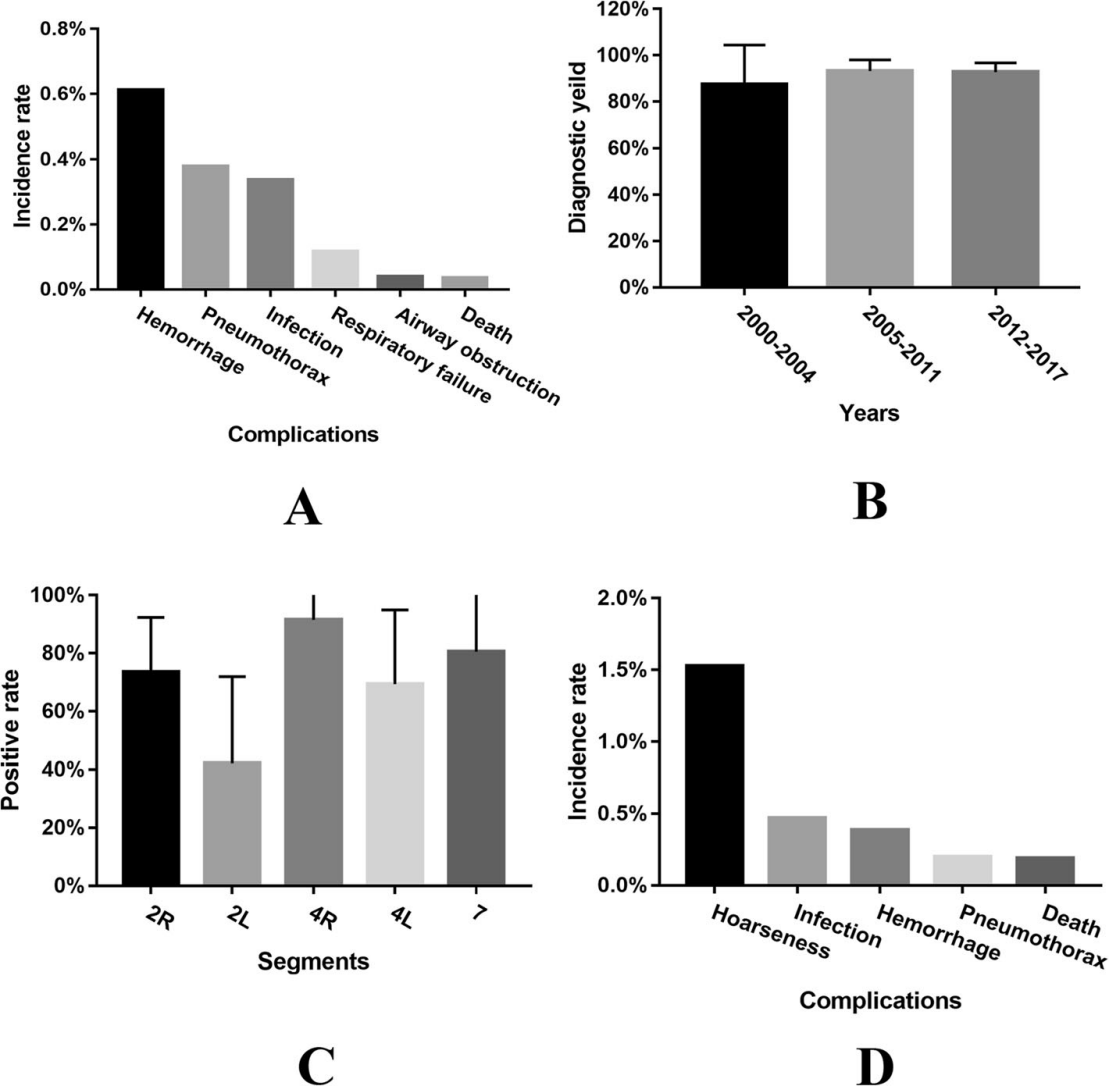
Analysis of clinical points regarding EBUS-TBNA and mediastinoscopy. **a** The top three complications following EBUS-TBNA were hemorrhage (53/8662:0.61%), pneumothorax (46/12432:0.37%) and infection (34/11250:0.30%), respectively. **b** The detection rate by using mediastinoscopy increased slightly. **c** The positive rate of 4^th^R (91.5% ± 9.35%) and 7th (80.56% ± 19.47%) lymph node by using mediastinoscopy were significantly higher than others (*P* < 0.05). **d** Hoarseness (67/4387:1.53%) was the top one complication following mediastinoscopy

The detection rate of lung node by EBUS-TBNA remained to be 86.06 ± 9.70%. The diagnostic sensitivity, specificity, accuracy, positive predictive value and negative predictive value of EBUS-TBNA for the mediastinal staging of lung cancer were 85.48% ± 12.89%, 99.09% ± 3.15%, 92.88% ± 4.99%, 98.70% ± 3.03%, 83.03% ± 15.46%, respectively. As shown in Fig. 2a, the top three complications following EBUS-TBNA were hemorrhage (53/8662:0.61%), pneumothorax (46/12432:0.37%) and infection (34/11250:0.30%), respectively. Four hemorrhage cases were administrated with further intervention with one perioperative death. The other 49 cases with mild hemorrhage were discharged for rehabilitation [32, 33]. In 46 pneumothorax cases, nine cases (9/46) and 37 cases (37/46) were administrated with closed drainage and conservative treatment, respectively [32–35]. Perioperative mortality was relatively low (4/11189:0.04%). Besides the above mentioned one case died of severe hemorrhage, there was one case died of cerebral infarction and two unexplained deaths [32, 33, 36].

### Mediastinoscopy: Biopsy of the lesion or lymph node in the vicinity of the trachea, the subcarinal and the bronchi area

The detection rate of lung cancer by mediastinoscopy increased slightly (Fig. 2b) which was reported to be 96% in recent years [37]. The diagnostic sensitivity, specificity, accuracy, positive predictive value and negative predictive value of mediastinoscopy for the mediastinal staging of lung cancer were 82.83% ± 10.63%, 100%, 93.98% ± 4.68%, 100%, 87.64% ± 13.00%, respectively. Intriguingly, the positive rates of 4^th^R (91.5% ± 9.35%) and 7th (80.56% ± 19.47%) lymph node were significantly higher than others (*P* = 0.03) (Fig. 2c). As shown in Fig. 2d, hoarseness (67/4387:1.53%) was the top one complication following mediastinoscopy. Among the abovementioned 67 cases with hoarseness, nine cases (9/67) suffered from permanent hoarseness, two cases (2/67) recovered partially by vocal cord medialization and six cases (6/67) recovered within a few months [37–45]. Perioperative mortality was relatively low (4/2137: 0.19%). The death causes among three cases were aortic laceration, stroke, and cardiac arrest, respectively, and one case die of unexplained cause [46].

### CTC: Biopsies of tumor cells shed from solid tumor lesion into peripheral blood

**Fig. 3:**
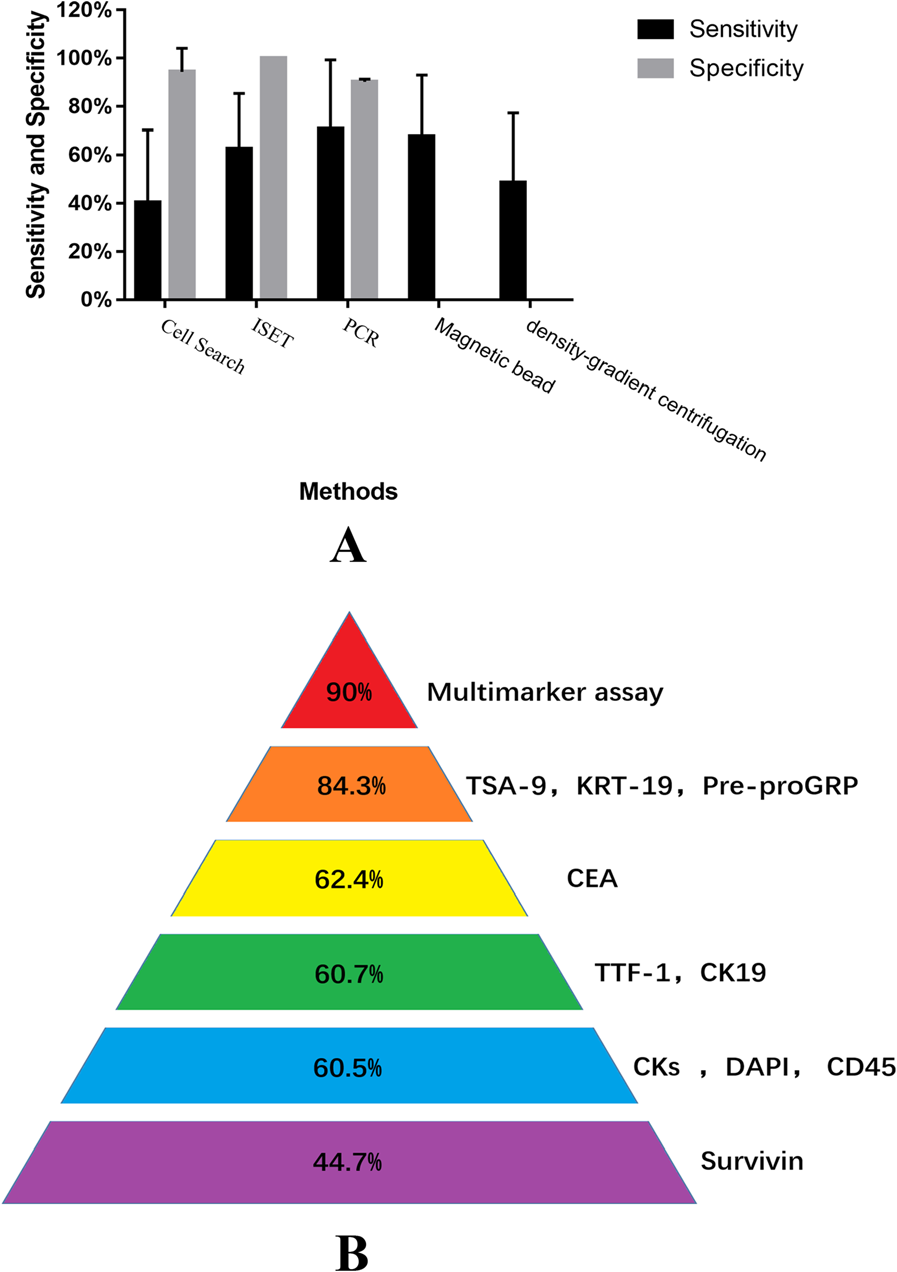
Analysis of clinical points regarding CTCs. **a** Sensitivity of PCR seemed to be highest (78.81 ± 14.72%). Specificity of ISET, PCR and Cell search was relatively high (100%, 90.88 ± 0.53% and 94.33 ± 9.82%). **b** Sensitivity of Multimarker assay seemed to be highest(90%) including 17 target genes: AGR2, CEACAM5, MMP11, STRN3, CEACAM6, COL5A2, AMPH, CEACAM7, ABCC3, THY1, COL6A3, ENO1, PNN, SCFD1, KDELR3, KIAA0391, TACSTD1

The mean sensitivities of a variety of methods to detect CTC remained to be 63.05%. As shown in Fig. 3a, sensitivity of PCR seemed to be highest (78.81 ± 14.72%). Sensitivity of Density-gradient, ISET and Magnetic bead seemed to be higher than 60% (71.32% ± 2.8%, 67.75% ± 21.22% and 67.85% ± 25.24%, respectively). Specificity of ISET, PCR and Cell search was relatively high (100%, 90.88 ± 0.53% and 94.33% ± 9.82%, respectively). There was no published data regarding specificity of Magnetic bead and density-gradient.

Intriguingly, there are a variety of biomarker combination for CTCs identification by using PCR yielding different sensitivities. As shown in Fig. 3b, the sensitivity of Multimarker assay seemed to be highest (90%). Besides, the sensitivity of the combination of TSA-9, KRT-19, Pre-proGRP was satisfactory (84.3%).

## Discussion

Considering the exquisite anatomy of the mediastinum, hilar and lung parenchyma, the equipment and technique, e.g., percutaneous lung biopsy, ENB, EBUS-TBNA, and Mediastinoscopy developed quickly. Furthermore, liquid biopsy, e.g., CTC detection has been introduced and a few pilot studies regarding early diagnosis of lung cancer have been published [47–65]. According to application in specific location and situation, we systemic reviewed clinical updates of these approaches focusing on development trends, detection rate and complications .

CT-guided PTNB is regarded as an effective and feasible procedure to detect a difficult nodule with advantage of accurate positioning and high detection accuracy. Nevertheless, once the lesion diameter is less than 2 cm or the needle path length is more than 8 cm, the detection rate will drop dramatically [4]. In addition, the lesions in the vicinity of mediastinum vessels are challengers to clinicians with regards to safety. Currently, ENB is developed for biopsy of the lesions in deep lung parenchyma or mediastinum.

ENB is recommended in patients with lesions in lung parenchyma difficult to reach with conventional bronchoscopy or CT-guided PTNB. The detection rate of ENB increased gradually probably due to improvement of software and hardware. Eberhardt et al. [20] found nodule location has been noted to be an important factor in diagnostic yield, e.g., the yields from the lower lobes were significantly lower (29%; *p* = 0.01). However, Jensen et al. [22] found lobar location of nodule did not affect the diagnostic yield (*p* = 0.59). Therefore, we systematically analyzed the results of six studies mentioning detection rate and nodule location [14, 20, 22, 27, 29, 66], and found that there seemed to be no association between them (*p* = 0.433). The highest incidence of complication is pneumothorax (5.2%). However, pneumothorax following ENB was reported to be unrelated with age or sex [16], accordant with our results. Intriguingly, the incidence of pneumothorax seemed to be significantly negatively correlated with nodule size, probably due to difficulties varying with the size. Additionally, there was no reported ENB associated death, proving that ENB is relatively safe.

Empirically, EBUS-TBNA is suitable for biopsy of lesion in subcarinal and bilateral hilar area. EBUS-TBNA is also well utilized in the peripheral area with radial probe EBUS and in conjunction with ENB. As EBUS-TBNA has relatively high false negative rates, especially at station 4R or 7 lymph node, mediastinoscopy is still required for patients with suspicious nodal disease in these stations [67]. Cytological samples are usually taken by EBUS-TBNA, however, larger histological tissue samples are possible to obtain by mediastinoscopy.

Mediastinoscopy is always recognized as the gold standard for surgical staging of lung cancer which is suitable for biopsy in the vicinity of the trachea, the subcarinal and the bronchi area. Especially, the positive rate of station 4R^th^ (91.5 ± 9.35%) and 7th (80.56 ± 19.47%) lymph node were significantly higher than other stations (Fig. 2c). Nevertheless, as mediastinoscopy is an invasive approach, the incidences of complications are relatively remarkable.

CTC is a kind of liquid biopsies of tumor cells shed from solid tumor lesions (primary foci and metastases) into peripheral blood. Although the mean sensitivities of CTC detection were not satisfactory, the convenience of this non-invasive method seems to be incomparable. Sensitivity of PCR remained to be highest (78.81% ± 14.72%) as compared with other methods. Intriguingly, the sensitivities of PCR varies with combined biomarkers. Expectedly, the sensitivity of combination of multimarkers assay is highest (90%). Furthermore, the specificity of the three methods, i.e., ISET, PCR and Cell search, was relatively high (100%, 90.88% ± 0.53% and 94.33% ± 9.82%, respectively). Currently, CTC can be used as an auxiliary diagnostic method to provide a higher detection rate.

## Conclusions

The biopsy approaches should be chosen according to a variety of location and situation of lesions. CT-guided PTNB is regarded as an effective and feasible procedure for biopsy in lung parenchyma, however, diagnostic accuracy and incidence of complications may be impacted by lesion size or needle path length. ENB has an advantage for biopsy of smaller and deeper lesions in lung parenchyma. ENB plus EBUS imaging can further improve the detection rate. EBUS-TBNA and mediastinoscopy can be recommended for the biopsy in lower and upper mediastinum, respectively. The former is relatively safer and the latter provides more tissue acquisition and better diagnostic yield of 4R and 7th lymph node. CTC detection can be considered for adjuvant diagnosis.

## Abbreviations

CTC: Circulating tumor cell
EBUS-TBNA: Endobroncheal Ultrasonography-Transbronchial Needle Aspitation
ENB: Electromagnetic navigation bronchoscopy
PCR: Polymerase chain reaction
PTNB: Percutaneous transthoracic needle biopsy
